# Combined biophysical and soluble factor modulation induces cardiomyocyte differentiation from human muscle derived stem cells

**DOI:** 10.1038/srep06614

**Published:** 2014-10-14

**Authors:** Jason Tchao, Lu Han, Bo Lin, Lei Yang, Kimimasa Tobita

**Affiliations:** 1 Department of Bioengineering, University of Pittsburgh, Pittsburgh, PA, USA; 2 Developmental Biology, University of Pittsburgh, Pittsburgh, PA, USA; 3 McGowan Institute of Regenerative Medicine, University of Pittsburgh, Pittsburgh, PA, USA

## Abstract

Cellular cardiomyoplasty has emerged as a novel therapy to restore contractile function of injured failing myocardium. Human multipotent muscle derived stem cells (MDSC) can be a potential abundant, autologous cell source for cardiac repair. However, robust conditions for cardiomyocyte (CM) differentiation are not well established for this cell type. We have developed a new method for CM differentiation from human MDSC that combines 3-dimensional artificial muscle tissue (AMT) culture with temporally controlled biophysical cell aggregation and delivery of 4 soluble factors (microRNA-206 inhibitor, IWR-1, Lithium Chloride, and BMP-4) (4F-AG-AMT). The 4F-AG-AMT displayed cardiac-like response to β-adrenergic stimulation and contractile properties. 4F-AG-AMT expressed major cardiac (NKX2-5, GATA4, TBX5, MEF2C) transcription factors and structural proteins. They also express cardiac gap-junction protein, connexin-43, similar to CMs and synchronized spontaneous calcium transients. These results highlight the importance of temporal control of biophysical and soluble factors for CM differentiation from MDSCs.

Developing cardiac and skeletal muscle share major transcription factors and structural proteins during development^1^. In vitro-based 3D artificial muscle tissues (AMT) made using skeletal muscle derived stem cells (MDSC) and induced pluripotent stem (iPS) cell derived cardiac progenitors also share these biochemical properties and are also functionally similar, suggesting that certain developmental similarities between cardiac and skeletal muscle are recapitulated using in vitro models^2^. Skeletal muscle and the heart are two potential sources of myogenic stem cells. The heart is a relatively small organ containing a resident population of cardiac stem cells that are difficult to isolate due to the heart's status as a vital organ. In contrast, skeletal muscle is the body's largest organ by mass, making it a large reservoir for myogenic stem cells, and stem cells can be isolated in clinically relevant numbers without significant risk to the patient's life. Thus, MDSCs from skeletal muscle are a promising stem cell source if they can be adapted to suit cardiac function.

Previous studies have shown that MDSCs are multipotent and less committed to the skeletal myogenic lineage than myoblasts^3^. Furthermore, in vivo cell transplantation studies have demonstrated increased donor cell survival, better engraftment, more robust resistance to oxidative stress, and improved cardiac function in animals receiving MDSCs compared to myoblasts (satellite cells)^4^. Undifferentiated MDSCs can also be transplanted as cell sheets without arrhythmias^5^, which were a major problem in early trials of skeletal myoblast transplantation^6^.

In order to achieve more positive therapeutic outcomes, the transplanted cells/tissues should resemble those which they are intended to replace. Following this biomimetic approach, MDSCs should be induced to differentiate into cells which are similar to cardiomyocytes (CM). Unfortunately, significant knowledge gaps still exist regarding how stem cells are regulated at the genetic and epigenetic levels and particularly how the cardiac and skeletal myogenic programs are tied to one another. The goal of the current study is to integrate biophysical and soluble factors which are known to promote cardiomyogenesis in order to improve AMT differentiation. To that end, we have introduced a combination of biophysical and soluble factor-based stimuli in a controlled manner in order to provide an integrated approach for induction of MDSCs into more functionally competent CM-like cells, which are capable of contraction and intercellular integration. We have identified a sequential treatment of four soluble factors, which, combined with optimized biophysical conditions, produced cardiac-like tissue capable of coordinated contractions, improved force generation properties, and better response to isoproterenol (ISP).

MicroRNAs (miRNAs) are small, non-coding RNAs averaging 22 nucleotides in length. They regulate gene expression at the transcriptional and post-transcriptional levels^7^. While many miRNAs are ubiquitously expressed, others are tissue specific. Several miRNAs, including miR-1, -133, -and -206, have been shown to be highly enriched in striated muscle and are termed myoMIRs^8^. Subsequent studies confirmed that miR-206 is primarily expressed in skeletal muscle. It plays a role in somite development, myogenesis, and fibre type specification^9^. One of the experimentally verified downstream targets of miR-206 is connexin-43 (GJA1)^10^. In the adult heart, GJA1 is the predominant gap junction protein in ventricular CMs^11^ and is responsible for proper electrical propagation. In skeletal muscle, GJA1 is required for proper myoblast alignment and fusion, after which GJA1 is rapidly downregulated^10^. It is believed that abnormal, prolonged expression of GJA1 in skeletal muscle can interfere with neuromuscular junction function^9^. However, expression of GJA1 is desirable for stem cells to be used in the heart^12^. The miR-206 inhibitor is a chemically modified, single stranded RNA produced by Invitrogen as “Anti-miR” that specifically binds to endogenous miR-206 and inhibits its activity. By inhibiting its activity, GJA1 gap junctions can be preserved and skeletal muscle differentiation and fusion could be abrogated in favour of a more cardiac-like phenotype (Supplementary Fig. 1).

Lithium Chloride (LiCl) is an ionic compound with high water solubility. Lithium compounds are commonly used as psychiatric medication^13^, suggesting that they can be safely used to treat cells in vitro. LiCl can activate Wnt signalling by inhibiting GSK-3β^14^. This allows for the accumulation of β-catenin and the activation of β-catenin dependent genes, including GJA1. LiCl treatment has been shown to enhance GJA1 gap junction expression in skeletal myoblasts^15^ and differentiating ES-cell derived CMs^16^. GSK-3β is also involved in other signalling pathways, so it may also have effects in addition to Wnt activation.

IWR-1 is a small molecule that acts as a Wnt pathway inhibitor via interaction with Axin^17^. The Wnt pathway plays a critical role during cardiac differentiation. Temporal control of Wnt signalling by small molecule agonists and antagonists produces highly pure cultures of CMs from human pluripotent stem cells^18^. Wnt signalling is active during early cardiac differentiation and later suppressed during commitment of early mesoderm to cardiac mesoderm in directed pluripotent stem cell differentiation protocols^19^ and in the embryo^20^. There is also evidence that Wnt inhibition can prevent hypertrophic myotube formation in skeletal muscle cells^21^. We reasoned that since MDSCs are already derived from adult mesoderm tissue, inhibition of Wnt at the onset of differentiation rather than later stages would aid in cardiac specification.

Bone morphogenic protein-4, BMP-4, is a cytokine of the TGF-β family that is involved in cardiac differentiation. BMP-4 can activate cardiac genes in combination with Wnt signals at the expense of other lineages such as skeletal muscle in mesenchymal stem cells^22^. The combination of small molecule Wnt inhibitors with BMP-4 can synergistically induce cardiac differentiation in iPS cells^23^. It has also been shown that Wnt and BMP signals activate distinct sets of cardiac transcription factors^24^, suggesting that both are required for efficient cardiac differentiation. BMP signals also have a role in regulating GJA1 expression in osteoblasts^25^, chondrocytes^26^, and neurons^27^. Although it has not been experimentally verified, this suggests that BMP may also have a role in gap junction regulation in muscle. Appropriate timing and dosage are also important, since these signals can have stage-dependent effects^28^. For example, higher dosages^29^ or sustained treatment^30^ can push cells toward the osteogenic lineage.

In the present study, we sought to address the dualistic physical and chemical nature of stem cell differentiation. We combined physical (aggregation and 3D culture) and chemical factors to create an artificially engineered cardiac tissue. Our approach is unique in that it takes advantage of the inherent myogenic capacity of the stem cell source and uses extrinsic factors to tune the cellular phenotype without forcibly modifying any genes. The resulting cells share a number of similarities with cardiomyocytes, although some differences remain. These results highlight the potential and current limitations, both in theory and practice, of cellular engineering.

## Results

In our initial studies, we tested 4 chemical factors (miR-206 inhibitor, IWR-1, LiCl, and BMP-4) individually and in combination for their ability to reduce skeletal myotube formation and stimulate connexin-43 (GJA1) gap junction formation. We found that the sequential addition of all 4 factors reduced myotube formation to the greatest extent and qualitatively suggested better gap junction formation (Supplementary Fig. 1a–b). At the outset, we found that addition of LiCl and BMP-4 at the initiation of differentiation (day 0) prevented muscle differentiation (Supplementary Fig. 1c). Therefore, we switched to a sequential treatment format. Thus, we selected the sequential addition condition for our subsequent engineered tissue studies.

### Temporal Effects of MDSC Aggregate Formation

**Figure 1:**
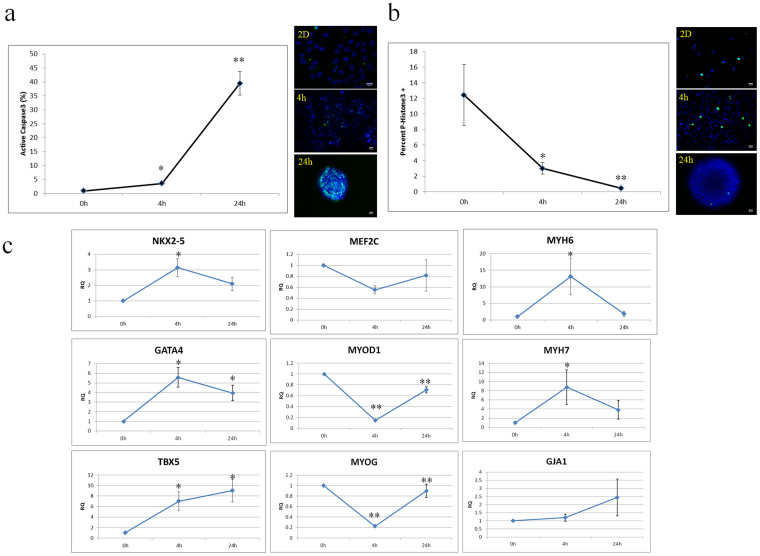
Temporal Effects of Aggregate Formation on MDSCs. (a) Aggregate formation markedly increased active caspase-3 expression (green) of MDSCs. (b) Aggregate formation significantly decreased MDSC phospho histone H3 (green) expression and proliferation. (c) Aggregate formation altered myocyte-specific gene expression patterns. * P < 0.05. ** P < 0.001 vs. baseline. Blue indicates nuclear DAPI staining. Scale bar indicates 20 μm.

First, we examined the effects of MDSC aggregate formation on cell death, proliferation, and differentiation. Cell aggregate formation is important for CM differentiation from stem cells. Embryonic stem (ES) cells are routinely aggregated into embryoid bodies and cultured in suspension using cardiac differentiation protocols for pluripotent stem cells^31^. It is believed that aggregate culture of stem cells recapitulates important physical aspects of embryonic development such as cell-cell interactions and can mediate processes including differentiation, proliferation, and apoptosis^32^. For example, aggregate formation kinetics can affect β-catenin signalling and subsequent differentiation through cadherin junctions^33^. Rotary orbital suspension culture is a common method to generate stem cell aggregates. In addition to cell-cell contacts, this method also introduces hydrodynamic shear, which can also alter gene expression^34^. Muscle derived stem cells cultured as 3D aggregates, termed myospheres, also display electrophysiological properties similar to CMs, which are modulated by culture conditions^35^. Aggregate culture can also significantly reduce proliferation^36^, and affect apoptosis^37^. In light of these multiple effects, aggregate culture conditions should be carefully selected to achieve the desired outcome. We noted that active caspase-3 expression increased significantly at 4 hours (3.6 ± 0.4%, n = 4 fields, p < 0.05) and 24 hours (39.5 ± 4.2%, n = 4, p < 0.001) of aggregate formation compared to 0 hours (0.9 ± 0.5%, n = 4) indicating that cells were entering an apoptotic fate at longer culture periods (Figure 1a). We also examined proliferation by phospho histone H3 expression and noted that proliferation decreased in a time dependent manner (Figure 1b) at 4 hours (3.1 ± 0.4%, n = 10, p < 0.05) and 24 hours (0.5 ± 0.1%, n = 10, p < 0.001) relative to actively proliferating cells on a standard tissue culture surface (12.4 ± 3.5%, n = 10). To examine differentiation in aggregates, we looked at muscle specific gene expression at different stages of aggregate formation (Figure 1c). Aggregate formation upregulated cardiac transcription factor (NKX2-5, GATA4, and TBX5) gene expression (n = 6). It also upregulated later cardiac differentiation markers MYH6 and MYH7 (n = 6). Interestingly, skeletal muscle transcription factors MYOD1 and MYOG were maximally suppressed at 4 hours but began to return to basal level by 24 hours (n = 6). To determine if MDSCs can differentiate into a muscle phenotype within aggregates, we cultured aggregates for 2 weeks in suspension culture in differentiation media (DM). We found that MDSCs within 2 week old aggregates stained positive for cardiac troponin-T (TNNT2, Data not shown.). However, a striated muscle pattern was not present, and the cells did not beat spontaneously. These results suggest that aggregate formation is important for triggering the switch from proliferation to differentiation and early activation of muscle gene expression. Short term aggregate formation appears to maintain cell viability and suggests CM-like differentiation. While MDSC aggregate formation is important for functional CM differentiation, long-term aggregate culture may not be the optimal environment for MDSCs.

### Effects of soluble factors on Gene and Protein Expression of AMT

**Figure 2:**
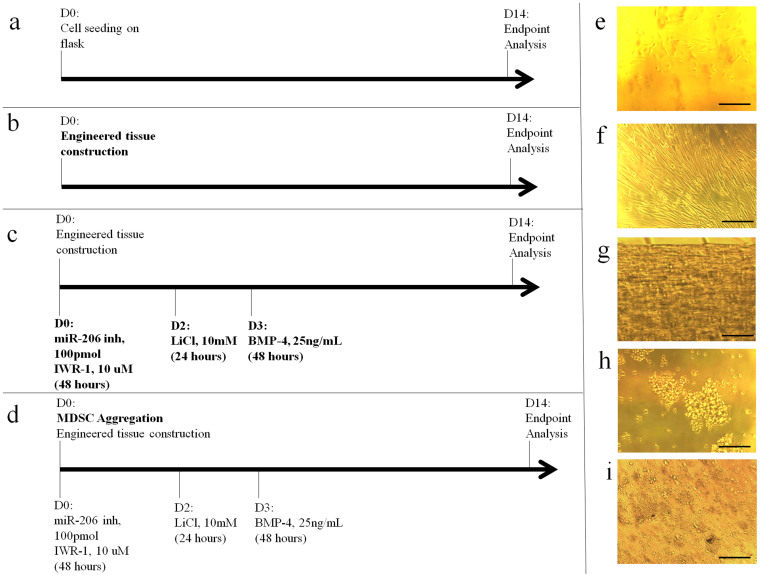
Differentiation of human MDSC. (a) 2D: MDSCs are seeded on a tissue culture flask at high density and allowed to differentiate. (b) AMT: MDSCs are cultured within 3D artificial muscle tissue. (c) 4F-AMT: MDSCs are cultured in 3D artificial muscle tissue and treated with 4 chemical factors. (d) 4F-AG-AMT: MDSC aggregates are cultured in 3D artificial muscle tissue and treated with 4 chemical factors. (e) Undifferentiated MDSCs on tissue culture flask. (f) 2D Differentiated MDSCs at day 14. (g) MDSCs in AMT. (h) MDSC aggregates after 4 hours of rotary suspension culture. (i) MDSC aggregates in AMT immediately after tissue construction. Scale bar indicates 250 μm.

**Figure 3:**
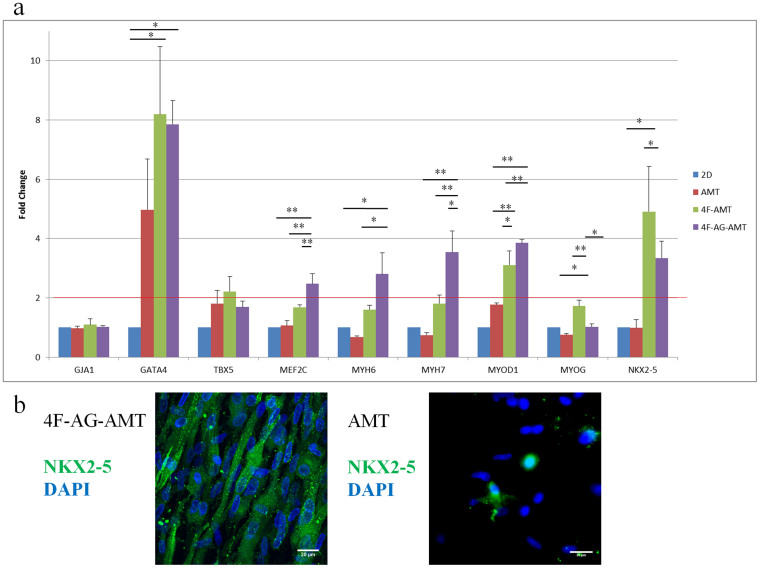
(a) Gene Expression of Day 14 Differentiated MDSCs. Muscle-specific gene expression of MDSCs is dependent on culture conditions. Values are expressed as fold change in gene expression and normalized to 2D differentiation protocol (2D). Red line indicates twofold change. *P < 0.05. **P < 0.001. (b) NKX2-5 protein expression in 4F-AG-AMT and AMT was detected by immunohistochemistry.

We examined gene expression of MDSCs differentiated under the 4 different protocols (Figure 2) of combined biophysical and soluble factors (Figure 3a). All genes were expressed regardless of the protocol used. 4F-AMT increased expression of cardiac transcription factors NKX2-5, GATA4, and to a lesser extent, TBX5 (n = 6). Aggregate formation combined with 4 factor treatment (4F-AG-AMT) increased MEF2C, MYH6, and MYH7 expression in addition to the aforementioned genes (n = 6). Interestingly, MYOD1 also increased under the 4F-AMT and 4F-AG-AMT protocols (n = 6). MYOG was also elevated in the 4F-AMT protocol (n = 6). GJA1 expression remained unchanged across the differentiation protocols (n = 6). These data indicate that human MDSCs express both cardiac and skeletal muscle genes, a characteristic shared with iPS cell derived cardiomyocytes and immature developing cardiac and skeletal muscle^1^,^2^. 4F-AG-AMT also expressed NKX2-5 protein by immunohistochemistry, while AMT culture alone induced limited expression with many cells remaining negative for NKX2-5 (Figure 3b).

**Figure 4:**
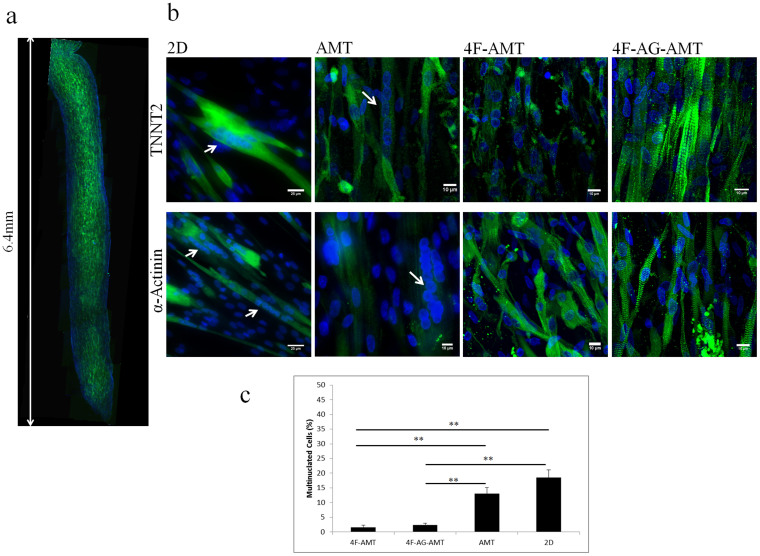
Histological Analysis of Day 14 Differentiated MDSCs. (a) Low magnification image of AMT showing α-Actinin expression throughout the construct, indicating MDSCs acquire a muscle phenotype. (b) Expression of α-Actinin and TNNT2 in MDSCs differentiated under various protocols. Scale bars indicate 10 μm or 20 μm as stated in each image. Arrows point to multinucleated cells. Blue indicates nuclear DAPI staining. (c) Percentage of multinucleated cells (n > 4 nuclei). 4-factor treatment significantly reduced the percentage of multinucleated cells. ** P < 0.001.

Histological assessments confirmed that MDSCs differentiate into a muscle phenotype in AMT (Figure 4a). MDSCs expressed α-actinin and TNNT2, both sarcomeric proteins, in all culture conditions (Figure 4b). However, a striated pattern was not clearly visible under 2D differentiation by either α-actinin or TNNT2 staining. MDSCs in 2D also formed elongated fibres containing many (more than 4) nuclei, which resemble skeletal myotubes (18.5 ± 2.5%, n = 18). In AMT, some cells retained the appearance of myotubes (13.1 ± 2.1%, n = 33). In AMT treated with 4 factors (4F-AMT [1.6 ± 0.6%, n = 38] and 4F-AG-AMT [2.4 ± 0.7%, n = 38]), myotubes were not readily apparent (Figure 4c). 4F-AG-AMT formed a muscle tissue with closely apposed muscle fibres that had a clear striated pattern by TNNT2 staining, which was less apparent in the 4F-AMT (no aggregate) group. These findings suggest that a combination of MDSC aggregate formation and 4 chemical factors was necessary for optimal differentiation towards a CM-like phenotype.

### Contractile Properties of AMT

**Figure 5:**
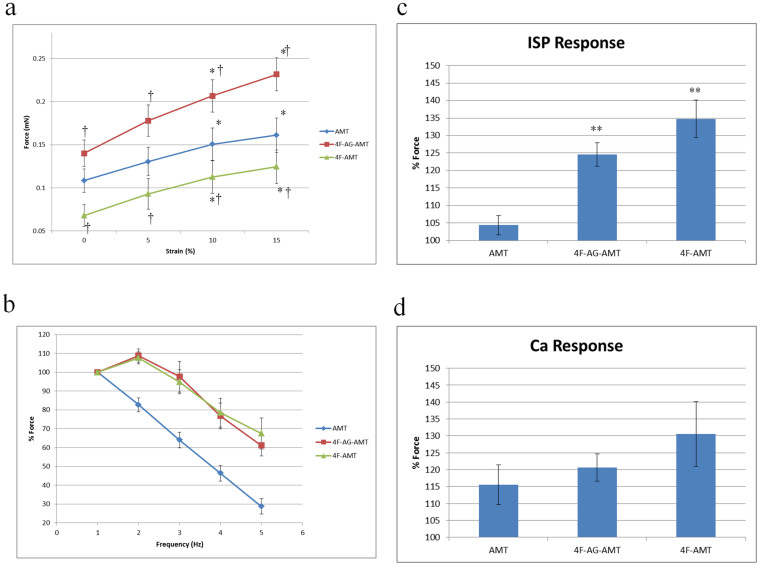
Contractile Properties of Artificial Muscle Tissue. (a) Force-strain relation of AMT. AMT increased force in response to increasing strain (Frank-Starling). * P < 0.05 vs. baseline. † P < 0.05 vs AMT. (b) Force-frequency relation of AMT at stimulation rates of 1–5 Hz. (c) Contractile response of AMT to isoproterenol. Values are expressed relative to pre-treatment values. ** P < 0.001 vs. AMT. (d) Contractile response of AMT to extracellular calcium. Calcium concentration of the bathing solution was increased from 2 mM to 5 mM.

All AMTs showed spontaneous beating activity from day 4, which gradually increased over the culture period and was sustained until day 14 (Supplementary Movie 1). We observed that the spontaneous beating activity of 4F-AG-AMT was visibly more vigorous than other groups. The presence of spontaneous beating activity confirms the differentiation of MDSCs into a functioning muscle phenotype. AMTs in all groups generated contractile force in response to electrical stimulation. The isometric force increased with strain (Frank-Starling mechanism) in all groups. 4F-AG-AMT (0.23 ± 0.02 mN at L_max_, n = 20) generated significantly higher force than AMT (0.16 ± 0.02 mN at L_max_, n = 16), and 4F-AMT generated less force than AMT (0.12 ± 0.02 mN at L_max_, n = 15) (Figure 5a). AMT showed a negative force-frequency relationship. In contrast, 4F-AMT and 4F-AG-AMT displayed a modestly positive force-frequency relationship at low frequencies (1–2 Hz) before becoming negative at higher stimulation frequencies (Figure 5b). 4F-AMT (134.7 ± 5.4%, n = 14, P < 0.001) and 4F-AG-AMT (124.6 ± 3.4% n = 24, P < 0.001) showed an increased positive inotropic response to isoproterenol compared to control untreated AMT (104.4 ± 2.8% n = 23, P < 0.001) (Figure 5c). All AMTs had a positive inotropic response to increased extracellular calcium, but there was no significant difference among groups (Figure 5d).

### Intercellular Coupling and Calcium Transients

**Figure 6:**
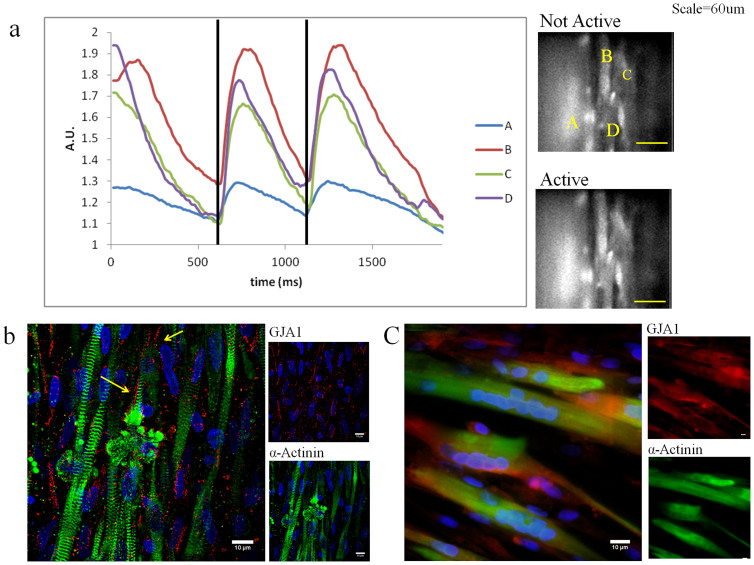
Intercellular Coupling of MDSCs. (a) Intracellular calcium transients of spontaneously beating MDSC-CMs recorded using Rhod-2 AM dye from 4 different locations. Trace shows that the transients begin and end in a synchronous manner (vertical lines). (b) Co-staining of 4-factor treated day 14 4F-AG-AMT with GJA1 (red), α-Actinin (green), and DAPI (blue). Arrows show gap junction formation between cells. Scale indicates 10 μm. (c) Co-staining of day 14 2D Differentiated MSDCs with GJA1 (red), α-Actinin (green), and DAPI (blue). Scale bar indicates 10 μm.

We assessed functional intercellular coupling by measuring intracellular calcium transients in AMT during spontaneous beating. AMT treated with 4 factors (4F-AMT and 4F-AG-AMT) showed that calcium transients in each cell were synchronous over the entire field of view (Figure 6a, Supplementary Movie 2). We co-stained cells for GJA1 and α-Actinin to identify gap junction intercellular coupling between differentiated myocytes. We observed a distinct dot-like pattern of connexin-43 staining in cells treated with 4 factors, which included concentrated staining at the border between adjacent cells (Figure 6b, yellow arrows). In contrast, cells that were differentiated under the 2D protocol stained positive for GJA1 and α-Actinin, but a distinct staining pattern was not observed (Figure 6c). Taken together, these data suggest that treatment of AMT with chemical factors can improve intercellular coupling.

**Figure 7:**
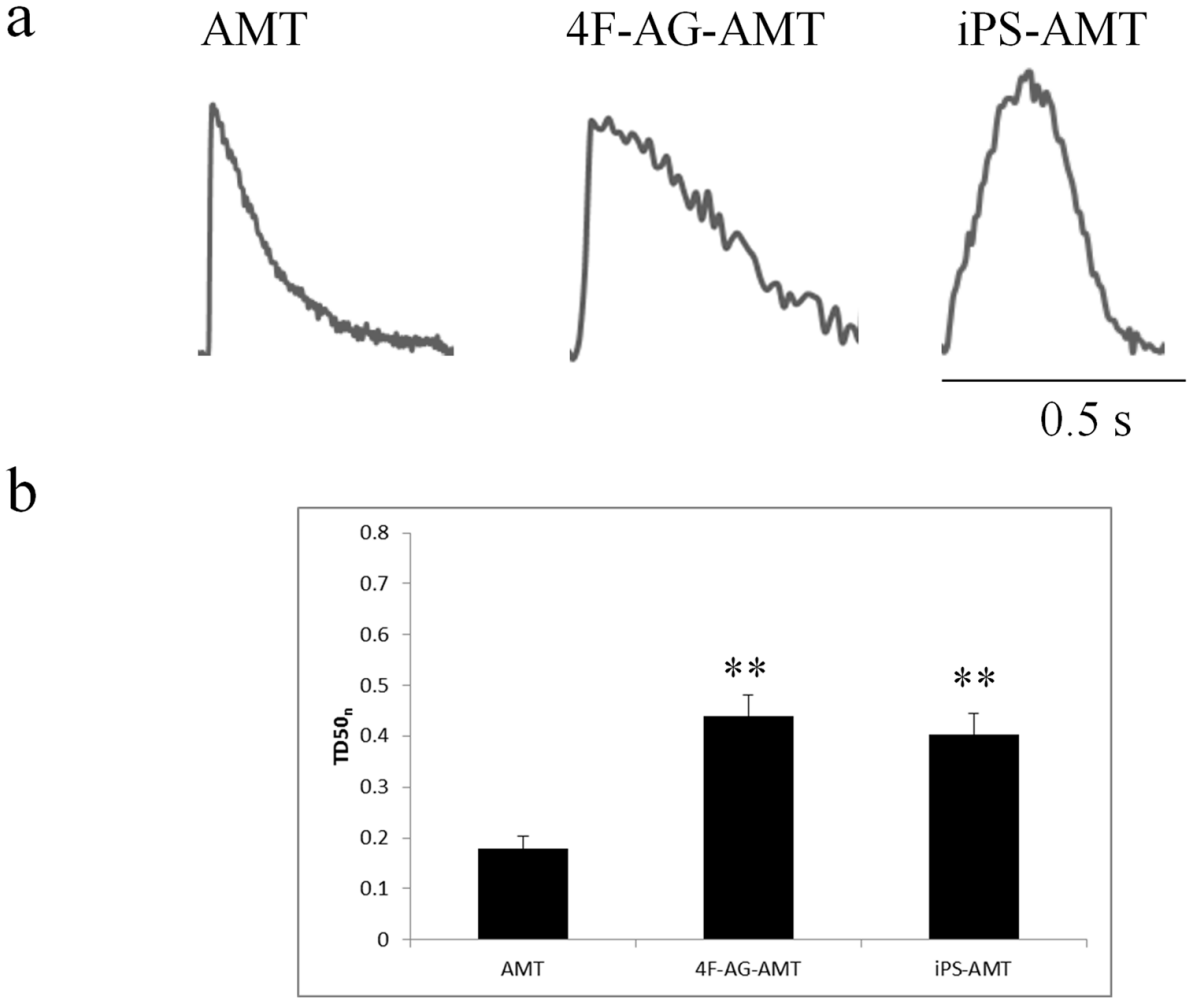
Calcium Transient Properties of AMT. (a) Representative spontaneous calcium transient waveforms from AMT, 4F-AG-AMT, and iPS-AMT. (b) Normalized calcium transient duration at 50% repolarization (TD50_n_) of AMT, 4F-AG-AMT, and iPS-AMT (n = 12 each). ** P < 0.001 versus AMT.

Next, we examined the waveforms of spontaneous calcium transients from AMTs (Figure 7a–b). Representative waveforms are shown in Figure 7a. Untreated AMTs showed a short, rapid transient pattern more characteristic of skeletal muscle (TD50_n_ 0.179 ± 0.023, n = 12). With treatment, 4F-AG-AMT shifted toward a slower transient (TD50_n_ 0.438 ± 0.044, n = 12) characteristic of CMs^22^ such as iPS cell derived CMs (TD50_n_ 0.403 ± 0.041, n = 12).

## Discussion

In the present study, we tested the hypothesis that timed soluble factor treatment and cell aggregate formation in conjunction with 3D culture could induce MDSCs to differentiate into CM-like cells as a functioning cardiac-like 3D tissue. While the full reconstitution of lost myocardial tissue by MDSCs, or any stem cell, remains more of an ideal than an attainable goal, our findings represent a stepwise advancement over existing cell therapy modalities.

The repair of predominantly non-regenerative organs such as the heart is the holy grail of regenerative medicine. Direct injection of stem cells and other cell types has yielded modest functional benefits through what are believed to be paracrine effects, which stimulate revascularization of the infarcted area, promote cell survival, and reduce scarring^38^. However, true regeneration requires remuscularization of the infarcted region. Despite recent advances, this goal remains elusive for the heart. Protocols exist to generate CMs from ES cells^39^, iPS cells^40^, and fibroblasts (direct reprogramming)^41^, but the use of these cells clinically is limited by safety and ethical issues. In the arena of adult stem cells, hematopoietic stem cells and bone marrow mesenchymal stem cells do not differentiate into muscle efficiently or require direct contact with CMs^42^, which are already depleted in the injured area^43^. True regeneration requires cells which can readily differentiate into a muscle phenotype. Stem or progenitor cells from cardiac and skeletal muscle tissue meet this criterion. However, cardiac stem cells require invasive isolation and are difficult to expand in vitro, and genetically unmodified skeletal myoblasts (committed satellite cells) have limited functional integration with host tissue due to differences in terminally differentiated cardiac and skeletal muscle^44^. MDSCs, given their plasticity and myogenic nature, are a promising cell source and may have the potential to differentiate into CM-like cells.

Realizing this goal requires recognition of the complexities of the stem cell niche and the factors that drive stem cell differentiation. Stem cell biology and tissue engineering have gradually moved away from traditional cell culture on a plastic dish and implemented novel approaches such as 3D bioreactors and cell aggregate culture in order to better recapitulate physiological conditions. In this study, we attempted to create a biomimetic environment for MDSC differentiation by providing an external environment containing collagen and other extracellular matrix factors (AMT) and dynamically regulating cell-cell interactions and soluble factor signalling by temporally controlling cell aggregate formation and treatment of signalling molecules. By using this integrative approach, we have successfully generated functional CM-like cells in vitro from human-origin MDSCs.

Multiple pathways regulate heart organogenesis including BMP, Wnt, and Notch signalling^45^. These signals are delivered in a temporally controlled manner, and the same signal can have opposing effects at different stages of development^20^,^46^,^47^. Reflecting this idea, we found in our initial studies that addition of LiCl or BMP-4 at day 0 inhibited muscle differentiation if they were added too early (Supplementary Fig. 1c). This is reasonable given that these pathways are also involved with maintenance or expansion of stem cell pools rather than differentiation in certain contexts^48^,^49^. However, they did not interfere with muscle differentiation if they were added sequentially after initial IWR-1 and miR-206 inhibitor treatment (Supplementary Fig. 1a). We attempted to mimic this temporal aspect in vitro. While these signals may not be fully representative of in vivo conditions, we were able to generate cells which are able to generate force as a functional muscle tissue while preserving their intercellular connectivity. miR-206 inhibition should theoretically inhibit skeletal muscle differentiation and prevent subsequent fusion. This combination of cytokines and small molecules with miR-206 inhibition blocked terminal skeletal muscle differentiation while simultaneously promoting CM differentiation. 4-factor treatment increased expression of cardiac transcription factors NKX2-5 and GATA4. 4F-AG-AMT also expressed NKX2-5 at the protein level. We observed both cytoplasmic and nuclear expression of NKX2-5. Although cardiac transcription factors such as NKX2-5 and GATA4 are typically present in the nucleus in mature CMs, they have also been reported to have a cytoplasmic component in some CM-like cell types^50^,^51^,^52^. Further studies are required to determine whether the NKX2-5 in MDSCs can shift toward the pattern seen in mature CMs. Visually, we observed a reduced incidence of fusion into myotubes in 4 factor treated AMTs. We also saw a connexin-43 distribution pattern similar to foetal CMs. With respect to function, we observed a cardiac-like force-frequency relationship with a small positive slope in the range of physiological human heartbeat similar to neonate CMs^53^. Without treatment, cells lose their distinct connexin staining pattern and show a diffuse cytoplasmic distribution. Since we did not observe a difference in GJA1 expression at the mRNA level, we surmise that GJA1 may be more stable than expected in differentiated MDSCs. The AMTs also showed an increased contractile response to isoproterenol, a cardiotropic compound. The waveform of 4 factor treated MDSC calcium transients also shifted away from the short duration exhibited by satellite cells and toward a slower cardiac-like pattern. Taken together, these data suggest that timed cytokine/small molecule treatment and transient miR-206 inhibition were sufficient to produce the desired phenotype.

Surprisingly, 4-factor treatment increased the expression of MYOD1, a skeletal muscle specific transcription factor. Although MYOD1 is generally considered a skeletal muscle specific factor, we have previously reported that MYOD1 is expressed at the gene level in the heart during murine development and is upregulated during differentiation of human iPS cells and rat MDSCs into CMs^1^,^2^. These findings have also been supported by studies in other species^54^. The role that MYOD1 may play during cardiac differentiation is unknown. Further studies are needed to elucidate its role. 4F-AMT also generated less force overall compared to AMT. This could be a result of differences in sarcomere length, which is proportional to force generation capacity. As cells differentiate into skeletal muscle, they elongate and some fuse, forming even larger fibres. However, the improvements in the force-frequency relationship and response to isoproterenol may reflect changes in excitation-contraction coupling and calcium handling at the cellular level.

MDSC aggregate formation had dynamic effects on cell proliferation, apoptosis, and differentiation. We previously used 24-hour aggregate formation time in our studies with rat MDSCs^36^. However, in our current study with human MDSCs, we found that a short period of aggregate formation was sufficient to trigger early cardiac differentiation markers and suppress skeletal muscle differentiation markers. Although the mechanisms by which cell-cell contact within aggregates influence cell differentiation are not well understood, it is evident that it plays an important role. One study found that aggregate formation can directly impact β-catenin signalling within embryoid bodies during cardiac differentiation^33^. Direct contact of mesenchymal stem cells with CMs is also a prerequisite for their differentiation into CMs^55^. Cell aggregation occurs in two stages: an initial stage of aggregate formation driven by integrin-ECM binding followed by a period of spheroid compaction driven by cadherin-cadherin interactions^56^. It is possible that downstream effectors of integrin-ECM binding can affect genes involved in CM differentiation. Cell death increased significantly in aggregates after 24 hours. Cell death may occur as a result of diffusion limitations of nutrients and oxygen or by hydrodynamic shear during rotary suspension culture. Proliferation also decreased in a time dependent manner. While differentiation is the end goal of this study, maintenance of proliferative activity is desirable for tissue formation, and foetal-like proliferative myocytes have better therapeutic benefit in vivo than more mature CMs^57^. We also tested long-term culture of MDSCs in aggregates to see if they could differentiate into CMs. After 2 weeks of culture as aggregates in suspension, MDSCs expressed TNNT2 protein. However, they did not beat spontaneously, and a striated pattern was not visible. These findings suggest that aggregate formation is important, but additional factors are required to trigger full differentiation of MDSCs into functional CMs.

Combined with AMT culture and 4-factor treatment, short term aggregate formation resulted in robust differentiation of MDSCs into functional myocytes. In addition to NKX2-5 and GATA4, 4F-AG-AMT upregulated cardiac transcription factor MEF2C and sarcomere related genes MYH6 and MYH7 compared to other groups. 4F-AG-AMT also showed good tissue formation and prominent striations by both TNNT2 and α-Actinin staining. In agreement with these results, we saw an overall increase in force generation in the 4F-AG-AMT group compared to other groups. These findings suggest that the 4F-AG-AMT protocol provided the best conditions for functional muscle differentiation, and the differentiated muscle cells were morphologically distinct from typical skeletal myotubes. In fact, human skeletal myotubes do not normally spontaneously contract in vitro^58^. Persistent, rhythmic spontaneous beating is further evidence of both functional differentiation and more cardiac-like behaviour. Embryoid bodies and other stem cell aggregate systems recapitulate elements of early embryonic development. However, as tissues develop into more complex structures, additional factors may be required to drive their development into functional tissues. AMT contains collagen and other extracellular matrix factors, which may be critical for functional differentiation of MDSCs.

Recent efforts in cardiac tissue engineering have focused on embryonic stem cells and induced pluripotent stem cells. While these cells may serve as useful models for developmental studies, toxicity testing, or drug screening, ethical issues and questions concerning their safety and genomic stability currently limit their clinical use^59^,^60^. Adult stem cells such as bone marrow and adipose derived mesenchymal stem cells can be differentiated into CMs by co-culture with CMs or treatment with 5-azacytidine^42^. However, the rate of differentiation into muscle is low, and functional characterization has been limited. These cells may also co-express cardiac and skeletal muscle genes^22^. To our knowledge, we are the first group to report the creation of engineered cardiac tissue from human origin adult stem cells in vitro with the ability to generate contractile force and coordinated intracellular calcium transients at the tissue level. The level of force generation of our cardiac construct is comparable to values reported by other groups using ES or iPS cell-derived CMs but less than that of native heart tissue^61^.

There are a number of limitations to the current study. We were unable to perform detailed electrophysiological measurements of AMTs beyond measurement of calcium transients. However, others have reported that MDSCs cultured as aggregates display electrophysiological properties similar to bona-fide CMs^35^. The effects of each of the soluble factors are not fully understood. We chose the timing of the 4 factors based on the combination that permitted functional muscle differentiation while preserving intercellular connectivity. Wnt and BMP signalling are required to activate distinct sets of cardiac transcription factors^24^. They may also have overlapping effects and cross-talk with cell aggregate formation and ECM. The idea of preconditioning adult stem cells with a cocktail of cardiogenic compounds to enhance their therapeutic performance has been tested in the C-CURE clinical trial^62^. It was found to be feasible and safe. Such lineage guided cell therapy could be further enhanced by introducing signals in a timed manner and combining them with 3D culture as we have done. Finally, MDSCs differentiated under our protocol continue to express a combination of cardiac and skeletal muscle markers. Additional factors such as physical (electrical stimulation, cyclic stretch) or chemical (miRNAs, cytokines) stimuli may be necessary to further increase differentiation and maturation of MDSCs towards the cardiac lineage.

4F-AG-AMT derived CM-like cells share a number of similarities with native CMs or established CM differentiation methods. At the gene level, they express a number of early and late cardiac markers such as MEF2C, GJA1, MYH6, and MYH7 at levels comparable to iPS cell derived CMs (Supplementary Figure 3)^2^. They also express major proteins such as NKX2-5, TNNT2, and α-Actinin. However, expression of NKX2-5, GATA4, and TBX5 is several orders less, although the transcripts are present. Nonetheless, the cells are still able to develop a functional contractile phenotype. It is possible that other related factors can contribute to the differentiation process. GATA5 and 6 may compensate for the loss of GATA4, as GATA4 deficient ES cells can still differentiate into CMs^63^, and multiple connexin isoforms are expressed in the developing heart before GJA1 becomes dominant^64^. Skin fibroblasts can be directly reprogrammed into CMs without forced NKX2-5 induction^41^. Cardiac fibroblasts express many cardiac genes, although they are significantly different from typical mature CMs^65^. Thus, cardiac gene expression alone may not be sufficient to indicate cardiac differentiation. On the other hand, absence (or low expression) of certain genes does not necessarily preclude CM differentiation, which is a complex process with multiple levels of regulation. From here, we expanded our characterization of 4F-AG-AMT. The contractile force of 4F-AG-AMT is about 20% of rat engineered foetal cardiac tissue^36^. The positive force-strain relationship, flat or slightly positive force-frequency relationship, and modest response to ISP are similar to immature CMs^53^. Our calcium transient analysis indicated that cells in 4F-AG-AMT are able to beat synchronously in a coordinated fashion. Border GJA1 staining also suggested the formation of gap junctions. Furthermore, the GJA1 staining pattern resembled foetal CMs rather than mature CMs. In contrast to mature CMs, some intracellular GJA1 was also detectable. The significance of this finding requires further investigation. Connexins are known to perform other functions in cells in addition to gap junction formation^66^. Other connexin isoforms may also contribute to cell-cell coupling, as the cells are still immature. Overall, the data indicate the adoption of a number of immature CM characteristics, although some differences remain.

In summary, we have built upon the established importance of AMT by temporally controlling direct cell contact and soluble signals, two of the mechanisms by which cells communicate. As a result, the engineered tissue showed better contractile properties and preserved intercellular connectivity. These findings highlight the importance of the temporal, physical, and soluble aspects of the cellular environment for functional cardiac tissue engineering.

## Methods

### Cell Culture

MDSCs of 3 different human subjects (from 10 to 30 years old) were purchased from Cook Myosite, Inc (SkMDC, Cat. #Sk-1111, Lots S01059-14M, P301052-17M, P101040-20M). The MDSCs are isolated by Cook Myosite using a pre-plate technique based on their variable adherence to collagen coated flasks as previously described^67^,^68^. MDSCs have been previously characterised as a heterogeneous population with the majority of cells expressing CD56 and CD146^2^,^69^. Cells were cultured in Cook Basal Media (Cook Myosite, Inc., Pittsburgh, PA, USA) supplemented with 10% Growth Supplement (Cook Myosite, Inc., Pittsburgh, PA, USA) and 1% Antibiotic-Antimycotic solution (AAS, Invitrogen) on collagen-I coated flasks. Cells were used for no more than 4 passages and maintained below 50% confluence prior to initiation of differentiation. Human S3 iPS cells were previously established in Lei Yang's lab from healthy fibroblasts^40^. iPS cells were maintained on mouse embryonic fibroblast (MEF) feeder layers with regular human embryonic stem cell medium containing 10 ng/mL FGF-2^39^. iPS cells were differentiated into cardiomyocytes using our previously established protocol^39^,^40^. All growth factors were from R&D systems. Following differentiation, day 20 embryoid bodies were dissociated using 0.05% trypsin (Invitrogen) and plated on Matrigel (BD Bioscience) coated 6 well plates in High-Glucose Dulbecco's Modified Eagle Medium (DMEM, Cellgro) supplemented with 10% Foetal Bovine Serum (FBS), L-Glutamine (1:100, Invitrogen), and Penicillin Streptomycin (1:100, Invitrogen)

### Artificial muscle tissue (AMT) Construction and MDSC Differentiation

Undifferentiated MDSCs underwent 4 different differentiation protocols. In the 2D differentiation protocol (2D, Figure 2a), undifferentiated MDSCs (Figure 2e) were plated at high density (40000 cells/cm^2^) and exposed to differentiation media (DM) consisting of Cook Basal Media supplemented with 5% growth supplement, 1% AAS, and human FGF-2 (5 ng/mL) for 14 days (Figure 2f). In the 3-dimensional artificial muscle tissue protocol (AMT, Figure 2b and Supplementary Fig. 2), MDSCs were cultured in DM within the 3D artificial muscle tissue (Figure 2g). To construct AMT, Liquid rat tail collagen type I (3 mg/mL) was neutralized with 0.1 N NaOH and mixed with Matrigel with a collagen: Matrigel ratio of 0.8 and a final collagen concentration of 0.67 mg/mL. Trypsinized MDSCs were seeded in a collagen/Matrigel mixture at a density of 0.5 million cells per construct using a Flexcell Tissue Train Culture system (FX-4000, Flexcell International) with a total volume of approximately 200 μL per construct to form a linear shaped construct. In the 4 factor artificial muscle tissue protocol (4F-AMT, Figure 2c), AMT was constructed as described, cultured in DM, and treated with 4 chemical factors: miR-206 inhibitor (100 pmol, Life Technologies), IWR-1 (10 uM), LiCl (10 mM), and BMP-4 (25 ng/ml) at different time points as illustrated in Figure 2c. For transfection of miR-206 inhibitor, X-treme Gene siRNA transfection reagent (Roche) was used according to the manufacturer's instructions. Using 100 pmol/construct, the majority of cells were transfected (~80% by Cy3 labelled small RNA, data not shown). In the MDSC aggregate 4 factor artificial muscle tissue protocol (4F-AG-AMT, Figure 2d), MDSCs were trypsinized, and MDSC-cell aggregates were obtained by rotation culture on a suspension culture plate at 50 rpm for 4 hours prior to tissue construction (Figure 2h)^36^. Aggregates were mixed with collagen and matrix factors (Figure 2i) to form a construct as previously described and treated with 4 chemical factors as illustrated in Figure 2d. 14 days was used as the end point for all protocols, and the media was changed every other day. For iPS cell cardiomyocyte derived artificial muscle tissue (iPS-AMT) construction, 0.5 million cardiomyocytes were mixed with the collagen/Matrigel mixture as described above and cast into a linear AMT.

### Real-time Polymerase Chain Reaction

Total RNA was prepared using Trizol solution and treated with TURBO DNA-free kit (Ambion, Austin, TX, USA). Primers, whose target genes are NKX2-5, GATA4, MEF2C, TBX5, GJA1, MYH6, MYH7, MYOD1, MYOG, and ACTB were obtained from Qiagen Quanti-Tect Primer Assay with the target fragment sizes approximately 100 base pairs. One step RT was performed using Applied Biosystems High Capacity RNA-to-cDNA Kit with a total of 2 μg RNA in a total volume of 20 μL with the following program: 37°C for 1 hour, 95°C for 5 minutes, 4°C hold. cDNA (1 μL) was used for RT- PCR using the following program: 50°C for 2 minutes, 95°C for 10 minutes. This was followed by 95°C for 15 seconds and 60°C for 1 minute, repeated for 40 cycles. The final stage was 95°C for 15 seconds, 60°C for 15 seconds. SYBR Green was used as a detector. 3 samples were run for each target gene per group from pooled samples using Applied Biosystems 7900HT system. Relative expression (RQ) was calculated using the *dd*Ct method with ACTB as an internal control.

### Immunohistochemical Staining

AMTs were fixed using 4% paraformaldehyde/PBS for 30 minutes and embedded in 13% polyacrylamide gel. 150 μm thick sections were made using a vibratory microtome and stained for cardiac troponin-T (TNNT2, MS-295-P, Thermo Scientific, 1:150 dilution), alpha sarcomeric actinin (α-Actinin, EA53, Sigma, 1:200 dilution), connexin-43 (GJA1, ab11369, Abcam, 1:100 dilution), active caspase-3 (ab2302, Abcam, 1:100 dilution), NKX2-5 (sc-8697, Santa Cruz, 1:50 dilution), and phospho histone H3 (P-histone3, ab32107, Abcam, 1:100 dilution) primary antibodies and Alexa Fluor secondary antibodies. Stained samples were scanned using a confocal microscope and used to generate 3D projection images of the tissue sections in ImageJ. Proliferation and apoptosis were assessed by calculating the percentage of total nuclei positive for phospho histone H3 and active caspase-3, respectively. Myotubes were identified as actinin positive cells containing 5 or more nuclei, since human CMs can contain up to 4 nuclei^70^.

### Mechanical Testing

The active force of AMTs was measured using a customized setup. Constructs were transferred to a muscle testing station perfused with warmed Ringer solution containing 2 mM CaCl_2_, 135 mM NaCl, 4 mM KCl, 10 mM Trizma-HCl, 8.3 mM Trizma-base, and 11 mM glucose. The constructs were attached to a force transducer (403A, Auroura Scientific, Auroura, Canada) using nylon meshes. The other end of each construct was attached to a micromanipulator. Field stimulation was applied using a stimulator at 5 ms duration, 100 V. The parameters were set at 10% above the threshold required to induce visible contraction of all constructs. The construct length was adjusted from 0% to 15% elongation. Force was measured at stimulation rates of 1–5 Hz and in response to isoproterenol (ISP, 1 μM) and extracellular calcium (Calcium Chloride, 5 mM).

### Intracellular Calcium Transient Measurement

Samples were loaded for 10 to 15 minutes at 37°C with Rhod 2-AM at a concentration of 5 μg/mL. After the dye was loaded, the samples were placed on the heated stage of a Leica (DM LFSA) microscope. Optical mapping was performed with high spatiotemporal resolution (64 × 64 pixels, 94 fps) at 37°C using a Hamamatsu EM-CCD camera (Model C9100-12). Calcium transients were recorded during spontaneous beating. Videos were processed in ImageJ (NIH) and analysed using densitometry techniques. A 3 × 3 pixel area was selected within each cell. The average pixel intensity within the region was calculated and normalized to the baseline resting value. TD50_n_ was calculated as the calcium transient duration at 50% repolarization and normalized to cycle length to correct for variations in beating rate.

### Statistical Procedures

One-way ANOVA was used to compare gene expression among experimental groups, proliferation rates, active caspase-3 expression, multinucleation percentages, changes in force in response to isoproterenol, and changes in force in response to extracellular calcium. A two-way repeated measures ANOVA was performed to compare active stress-strain relations. A post-hoc Tukey test was used to determine individual differences between experimental groups. Data were expressed as mean ± standard error. Statistical significance was defined by an unadjusted value of *P* < 0.05. A Bonferroni correction was applied when necessary in the case of multiple testing. All calculations were performed using Sigma Stat 3.0 software (Systat Software Inc.).

## Author Contributions

K.T. and L.Y. provided reagents for the experiments. J.T.and K.T. conceived and designed the experiments. J.T., L.H., B.L. and K.T. performed the experiments. J.T., L.H., B.L., L.Y. and K.T. analyzed the data and wrote the manuscript. K.T. approved the final version of the manuscript.

## Supplementary Material

Supplementary Information Supplementary Information

Supplementary Information Supplementary Movie 1

Supplementary Information Supplementary Movie 2

## References

[b1] Clause K. C. *et al.* Developing cardiac and skeletal muscle share fast-skeletal myosin heavy chain and cardiac troponin-I expression. PloS one 7, e40725, 10.1371/journal.pone.0040725 (2012). 22808244 10.1371/journal.pone.0040725 PMC3393685

[b2] Tchao J. *et al.* Engineered Human Muscle Tissue from Skeletal Muscle Derived Stem Cells and Induced Pluripotent Stem Cell Derived Cardiac Cells. Int J Tissue Eng 2013, 198762, 10.1155/2013/198762 (2013). 24734224 10.1155/2013/198762 PMC3984572

[b3] Usas A. & Huard J. Muscle-derived stem cells for tissue engineering and regenerative therapy. Biomaterials 28, 5401–5406, 10.1016/j.biomaterials.2007.09.008 (2007). 17915311 10.1016/j.biomaterials.2007.09.008 PMC2095130

[b4] Oshima H. *et al.* Differential myocardial infarct repair with muscle stem cells compared to myoblasts. Mol Ther 12, 1130–1141, 10.1016/j.ymthe.2005.07.686 (2005). 16125468 10.1016/j.ymthe.2005.07.686

[b5] Sekiya N. *et al.* Muscle-derived stem cell sheets support pump function and prevent cardiac arrhythmias in a model of chronic myocardial infarction. Mol Ther 21, 662–669, 10.1038/mt.2012.266 (2013). 23319053 10.1038/mt.2012.266 PMC3589171

[b6] Menasche P. Cardiac cell therapy: lessons from clinical trials. J Mol Cell Cardiol 50, 258–265, 10.1016/j.yjmcc.2010.06.010 (2011). 20600097 10.1016/j.yjmcc.2010.06.010

[b7] McCarthy J. J. The MyomiR network in skeletal muscle plasticity. Exerc Sport Sci Rev 39, 150–154, 10.1097/JES.0b013e31821c01e1 (2011). 21467943 10.1097/JES.0b013e31821c01e1 PMC4871711

[b8] Malizia A. P. & Wang D. Z. MicroRNAs in cardiomyocyte development. Wiley Interdiscip Rev Syst Bio Med 3, 183–190, 10.1002/wsbm.111 (2011). 21305703 10.1002/wsbm.111 PMC3058499

[b9] McCarthy J. J. MicroRNA-206: the skeletal muscle-specific myomiR. Biochimica et biophysica acta 1779, 682–691, 10.1016/j.bbagrm.2008.03.001 (2008). 18381085 10.1016/j.bbagrm.2008.03.001 PMC2656394

[b10] Anderson C., Catoe H. & Werner R. MIR-206 regulates connexin43 expression during skeletal muscle development. Nucleic Acids Res 34, 5863–5871, 10.1093/nar/gkl743 (2006). 17062625 10.1093/nar/gkl743 PMC1635318

[b11] Darrow B. J., Laing J. G., Lampe P. D., Saffitz J. E. & Beyer E. C. Expression of multiple connexins in cultured neonatal rat ventricular myocytes. Circ Res 76, 381–387 (1995). 7859384 10.1161/01.res.76.3.381

[b12] Roell W. *et al.* Engraftment of connexin 43-expressing cells prevents post-infarct arrhythmia. Nature 450, 819–824, 10.1038/nature06321 (2007). 18064002 10.1038/nature06321

[b13] Schou M. Lithium in psychiatric therapy and prophylaxis. J Psychiatr Res 6, 67–95 (1968). 4876759 10.1016/0022-3956(68)90047-2

[b14] Klein P. S. & Melton D. A. A molecular mechanism for the effect of lithium on development. Proc Natl Acad Sci USA 93, 8455–8459 (1996). 8710892 10.1073/pnas.93.16.8455 PMC38692

[b15] Du W. J., Li J. K., Wang Q. Y., Hou J. B. & Yu B. Lithium chloride preconditioning optimizes skeletal myoblast functions for cellular cardiomyoplasty in vitro via glycogen synthase kinase-3beta/beta-catenin signaling. Cells Tissues Organs 190, 11–19, 10.1159/000167699 (2009). 18957842 10.1159/000167699

[b16] Czyz J., Guan K., Zeng Q. & Wobus A. M. Loss of beta 1 integrin function results in upregulation of connexin expression in embryonic stem cell-derived cardiomyocytes. Int J Dev Biol 49, 33–41, 10.1387/ijdb.041835jc (2005). 15744665 10.1387/ijdb.041835jc

[b17] Chen B. *et al.* Small molecule-mediated disruption of Wnt-dependent signaling in tissue regeneration and cancer. Nat Chem Biol 5, 100–107, 10.1038/nchembio.137 (2009). 19125156 10.1038/nchembio.137 PMC2628455

[b18] Lian X. *et al.* Robust cardiomyocyte differentiation from human pluripotent stem cells via temporal modulation of canonical Wnt signaling. Proc Natl Acad Sci USA 109, E1848–1857, 10.1073/pnas.1200250109 (2012). 22645348 10.1073/pnas.1200250109 PMC3390875

[b19] Ueno S. *et al.* Biphasic role for Wnt/beta-catenin signaling in cardiac specification in zebrafish and embryonic stem cells. Proc Natl Acad Sci USA 104, 9685–9690, 10.1073/pnas.0702859104 (2007). 17522258 10.1073/pnas.0702859104 PMC1876428

[b20] Naito A. T. *et al.* Developmental stage-specific biphasic roles of Wnt/beta-catenin signaling in cardiomyogenesis and hematopoiesis. Proc Natl Acad Sci USA 103, 19812–19817, 10.1073/pnas.0605768103 (2006). 17170140 10.1073/pnas.0605768103 PMC1750922

[b21] Han X. H., Jin Y. R., Seto M. & Yoon J. K. A WNT/beta-catenin signaling activator, R-spondin, plays positive regulatory roles during skeletal myogenesis. J Biol Chem 286, 10649–10659, 10.1074/jbc.M110.169391 (2011). 21252233 10.1074/jbc.M110.169391 PMC3060516

[b22] Grajales L., Garcia J. & Geenen D. L. Induction of cardiac myogenic lineage development differs between mesenchymal and satellite cells and is accelerated by bone morphogenetic protein-4. J Mol Cell Cardiol 53, 382–391, 10.1016/j.yjmcc.2012.06.003 (2012). 22709559 10.1016/j.yjmcc.2012.06.003 PMC3426454

[b23] Ren Y. *et al.* Small molecule Wnt inhibitors enhance the efficiency of BMP-4-directed cardiac differentiation of human pluripotent stem cells. J Mol Cell Cardiol 51, 280–287, 10.1016/j.yjmcc.2011.04.012 (2011). 21569778 10.1016/j.yjmcc.2011.04.012 PMC3334336

[b24] Klaus A. *et al.* Wnt/beta-catenin and Bmp signals control distinct sets of transcription factors in cardiac progenitor cells. Proc Natl Acad Sci USA 109, 10921–10926, 10.1073/pnas.1121236109 (2012). 22711842 10.1073/pnas.1121236109 PMC3390862

[b25] Chatterjee B. *et al.* BMP regulation of the mouse connexin43 promoter in osteoblastic cells and embryos. Cell Communi Adhes 10, 37–50 (2003). 10.1080/15419060302064 12881039

[b26] Zhang W., Green C. & Stott N. S. Bone morphogenetic protein-2 modulation of chondrogenic differentiation in vitro involves gap junction-mediated intercellular communication. J Cell Physiol 193, 233–243, 10.1002/jcp.10168 (2002). 12385001 10.1002/jcp.10168

[b27] Bani-Yaghoub M., Felker J. M., Sans C. & Naus C. C. The effects of bone morphogenetic protein 2 and 4 (BMP2 and BMP4) on gap junctions during neurodevelopment. Exp Neurol 162, 13–26, 10.1006/exnr.2000.7294 (2000). 10716885 10.1006/exnr.2000.7294

[b28] Kattman S. J. *et al.* Stage-specific optimization of activin/nodal and BMP signaling promotes cardiac differentiation of mouse and human pluripotent stem cell lines. Cell Stem Cell 8, 228–240, 10.1016/j.stem.2010.12.008 (2011). 21295278 10.1016/j.stem.2010.12.008

[b29] Katagiri T. *et al.* Bone Morphogenetic Protein-2 Converts the Differentiation Pathway of C2c12 Myoblasts into the Osteoblast Lineage. J Cell Biol 127, 1755–1766, 10.1083/jcb.127.6.1755 (1994). 7798324 10.1083/jcb.127.6.1755 PMC2120318

[b30] Li G. *et al.* Differential effect of BMP4 on NIH/3T3 and C2C12 cells: implications for endochondral bone formation. J Bone Miner Res 20, 1611–1623, 10.1359/JBMR.050513 (2005). 16059633 10.1359/JBMR.050513

[b31] Sargent C. Y., Berguig G. Y. & McDevitt T. C. Cardiomyogenic Differentiation of Embryoid Bodies Is Promoted by Rotary Orbital Suspension Culture. Tissue Eng Pt A 15, 331–342, 10.1089/ten.tea.2008.0145 (2009). 10.1089/ten.tea.2008.0145 19193130

[b32] Carpenedo R. L., Sargent C. Y. & McDevitt T. C. Rotary suspension culture enhances the efficiency, yield, and homogeneity of embryoid body differentiation. Stem Cells 25, 2224–2234, 10.1634/stemcells.2006-0523 (2007). 17585171 10.1634/stemcells.2006-0523

[b33] Kinney M. A., Sargent C. Y. & McDevitt T. C. Temporal modulation of beta-catenin signaling by multicellular aggregation kinetics impacts embryonic stem cell cardiomyogenesis. Stem Cells Dev 22, 2665–2677, 10.1089/scd.2013.0007 (2013). 23767804 10.1089/scd.2013.0007 PMC3780328

[b34] Kinney M. A., Sargent C. Y. & McDevitt T. C. The multiparametric effects of hydrodynamic environments on stem cell culture. Tissue Eng Pt B Rev 17, 249–262, 10.1089/ten.TEB.2011.0040 (2011). 10.1089/ten.teb.2011.0040 PMC3142632 21491967

[b35] Poulet C., Wettwer E., Christ T. & Ravens U. Skeletal muscle stem cells propagated as myospheres display electrophysiological properties modulated by culture conditions. J Mol Cell Cardiol 50, 357–366, 10.1016/j.yjmcc.2010.10.011 (2011). 20971120 10.1016/j.yjmcc.2010.10.011

[b36] Clause K. C. *et al.* A three-dimensional gel bioreactor for assessment of cardiomyocyte induction in skeletal muscle-derived stem cells. Tissue Eng Pt C Methods 16, 375–385, 10.1089/ten.TEC.2009.0098 (2010). 10.1089/ten.tec.2009.0098 PMC2945363 19601695

[b37] Korff T. & Augustin H. G. Integration of endothelial cells in multicellular spheroids prevents apoptosis and induces differentiation. J Cell Biol 143, 1341–1352 (1998). 9832561 10.1083/jcb.143.5.1341 PMC2133072

[b38] Hassan N., Tchao J. & Tobita K. Concise review: skeletal muscle stem cells and cardiac lineage: potential for heart repair. Stem Cells Transl Med 3, 183–193, 10.5966/sctm.2013-0122 (2014). 24371329 10.5966/sctm.2013-0122 PMC3925055

[b39] Yang L. *et al.* Human cardiovascular progenitor cells develop from a KDR+ embryonic-stem-cell-derived population. Nature 453, 524–528, 10.1038/nature06894 (2008). 18432194 10.1038/nature06894

[b40] Lin B. *et al.* High-purity enrichment of functional cardiovascular cells from human iPS cells. Cardiovasc Res 95, 327–335, 10.1093/cvr/cvs185 (2012). 22673369 10.1093/cvr/cvs185 PMC4415083

[b41] Inagawa K. & Ieda M. Direct reprogramming of mouse fibroblasts into cardiac myocytes. J Cardiovasc Transl Res 6, 37–45, 10.1007/s12265-012-9412-5 (2013). 23054660 10.1007/s12265-012-9412-5

[b42] Koninckx R. *et al.* Human bone marrow stem cells co-cultured with neonatal rat cardiomyocytes display limited cardiomyogenic plasticity. Cytotherapy 11, 778–792, 10.3109/14653240902988818 (2009). 19878064 10.3109/14653240902988818

[b43] Tchao J. & Tobita K. Perspectives: Cardiomyocytes from Skeletal Muscle Stem Cells for Cardiac Repair. JSM Regen Med (2013).

[b44] Reinecke H., Poppa V. & Murry C. E. Skeletal muscle stem cells do not transdifferentiate into cardiomyocytes after cardiac grafting. J Mol Cell Cardiol 34, 241–249 (2002). 11851363 10.1006/jmcc.2001.1507

[b45] Rochais F., Mesbah K. & Kelly R. G. Signaling pathways controlling second heart field development. Circ Res 104, 933–942, 10.1161/CIRCRESAHA.109.194464 (2009). 19390062 10.1161/CIRCRESAHA.109.194464

[b46] Klaus A., Saga Y., Taketo M. M., Tzahor E. & Birchmeier W. Distinct roles of Wnt/beta-catenin and Bmp signaling during early cardiogenesis. Proc Natl Acad Sci USA 104, 18531–18536, 10.1073/pnas.0703113104 (2007). 18000065 10.1073/pnas.0703113104 PMC2141811

[b47] Tzahor E. Wnt/β-catenin signaling and cardiogenesis: timing does matter. Dev cell 13, 10–13 (2007). 17609106 10.1016/j.devcel.2007.06.006

[b48] Black B. L. in Sem Cell Dev Biol. 67–76 (Elsevier).

[b49] Friedrichs M. *et al.* BMP signaling balances proliferation and differentiation of muscle satellite cell descendants. BMC Cell Biol 12, 26, 10.1186/1471-2121-12-26 (2011). 21645366 10.1186/1471-2121-12-26 PMC3149017

[b50] Kim T. K. *et al.* Transcriptome transfer provides a model for understanding the phenotype of cardiomyocytes. Proc Natl Acad Sci USA 108, 11918–11923, 10.1073/pnas.1101223108 (2011). 21730152 10.1073/pnas.1101223108 PMC3141937

[b51] Xiang G. *et al.* Lentivirus-mediated Wnt11 gene transfer enhances Cardiomyogenic differentiation of skeletal muscle-derived stem cells. Mol Ther 19, 790–796, 10.1038/mt.2011.5 (2011). 21304494 10.1038/mt.2011.5 PMC3070113

[b52] Winitsky S. O. *et al.* Adult murine skeletal muscle contains cells that can differentiate into beating cardiomyocytes in vitro. PLoS Biol 3, e87, 10.1371/journal.pbio.0030087 (2005). 15757365 10.1371/journal.pbio.0030087 PMC1064849

[b53] Turnbull I. C. *et al.* Advancing functional engineered cardiac tissues toward a preclinical model of human myocardium. FASEB J 28, 644–654, 10.1096/fj.13-228007 (2014). 24174427 10.1096/fj.13-228007 PMC3898643

[b54] Chen X. *et al.* Tissue specific expression of Pax3/7 and MyoD in adult duck tissues. J Appl Anim Res 40, 284–288, 10.1080/09712119.2012.672311 (2012).

[b55] Rangappa S., Entwistle J. W., Wechsler A. S. & Kresh J. Y. Cardiomyocyte-mediated contact programs human mesenchymal stem cells to express cardiogenic phenotype. J Thorac Cardiovasc Surg 126, 124–132 (2003). 12878947 10.1016/s0022-5223(03)00074-6

[b56] Lin R. Z. & Chang H. Y. Recent advances in three-dimensional multicellular spheroid culture for biomedical research. Biotechnol J 3, 1172–1184, 10.1002/biot.200700228 (2008). 18566957 10.1002/biot.200700228

[b57] Fujimoto K. L. *et al.* Engineered fetal cardiac graft preserves its cardiomyocyte proliferation within postinfarcted myocardium and sustains cardiac function. Tissue Eng Pt A 17, 585–596, 10.1089/ten.TEA.2010.0259 (2011). 10.1089/ten.tea.2010.0259 PMC3043979 20868205

[b58] Guo X. *et al.* In vitro Differentiation of Functional Human Skeletal Myotubes in a Defined System. Biomater Sci 2, 131–138, 10.1039/C3BM60166H (2014). 24516722 10.1039/C3BM60166H PMC3917571

[b59] Martins-Taylor K. & Xu R. H. Concise review: Genomic stability of human induced pluripotent stem cells. Stem Cells 30, 22–27, 10.1002/stem.705 (2012). 21823210 10.1002/stem.705

[b60] Lefort N., Perrier A. L., Laabi Y., Varela C. & Peschanski M. Human embryonic stem cells and genomic instability. Regen Med 4, 899–909, 10.2217/rme.09.63 (2009). 19903007 10.2217/rme.09.63

[b61] Zhang D. *et al.* Tissue-engineered cardiac patch for advanced functional maturation of human ESC-derived cardiomyocytes. Biomaterials 34, 5813–5820, 10.1016/j.biomaterials.2013.04.026 (2013). 23642535 10.1016/j.biomaterials.2013.04.026 PMC3660435

[b62] Aalst C. & Genk L. Cardiopoietic Stem Cell Therapy in Heart Failure. J Am Coll Cardiol 61 (2013). 10.1016/j.jacc.2013.02.071 23583246

[b63] Narita N., Bielinska M. & Wilson D. B. Cardiomyocyte differentiation by GATA-4-deficient embryonic stem cells. Development 124, 3755–3764 (1997). 9367431 10.1242/dev.124.19.3755

[b64] Sohl G. & Willecke K. Gap junctions and the connexin protein family. Cardiovasc Res 62, 228–232, 10.1016/j.cardiores.2003.11.013 (2004). 15094343 10.1016/j.cardiores.2003.11.013

[b65] Furtado M. B. *et al.* Cardiogenic genes expressed in cardiac fibroblasts contribute to heart development and repair. Circ Res 114, 1422–1434, 10.1161/CIRCRESAHA.114.302530 (2014). 24650916 10.1161/CIRCRESAHA.114.302530 PMC4083003

[b66] Dbouk H. A., Mroue R. M., El-Sabban M. E. & Talhouk R. S. Connexins: a myriad of functions extending beyond assembly of gap junction channels. Cell Commun Signal: CCS 7, 4, 10.1186/1478-811X-7-4 (2009). 10.1186/1478-811X-7-4 PMC2660342 19284610

[b67] Qu-Petersen Z. *et al.* Identification of a novel population of muscle stem cells in mice: potential for muscle regeneration. J Cell Biol 157, 851–864, 10.1083/jcb.200108150 (2002). 12021255 10.1083/jcb.200108150 PMC2173424

[b68] Gharaibeh B. *et al.* Isolation of a slowly adhering cell fraction containing stem cells from murine skeletal muscle by the preplate technique. Nat Protoc 3, 1501–1509, 10.1038/nprot.2008.142 (2008). 18772878 10.1038/nprot.2008.142

[b69] Zheng B. *et al.* Isolation of myogenic stem cells from cultures of cryopreserved human skeletal muscle. Cell Transplant 21, 1087–1093, 10.3727/096368912X636876 (2012). 22472558 10.3727/096368912X636876 PMC4356195

[b70] Botting K. J. *et al.* Early origins of heart disease: low birth weight and determinants of cardiomyocyte endowment. Clin Exp Pharmacol Physiol 39, 814–823, 10.1111/j.1440-1681.2011.05649.x (2012). 22126336 10.1111/j.1440-1681.2011.05649.x

