# Supplementary material for: Combined biophysical and soluble factor modulation induces cardiomyocyte differentiation from human muscle derived stem cells
Source: Sci Rep. 2014 Oct 14;4:6614. doi: 10.1038/srep06614 (PMC4196107; doi:10.1038/srep06614)
Supplement: Supplementary Information [file srep06614-s1.pdf]

Supplementary Information

Combined biophysical and soluble factor modulation induces cardiomyocyte differentiation from human muscle derived stem cells

Jason Tchao[1], Lu Han[2], Bo Lin[2], Lei Yang[2], Kimimasa Tobita[1,2,3,*]

Department of Bioengineering[1], Developmental Biology[2], McGowan Institute of Regenerative Medicine[3], University of Pittsburgh, Pittsburgh, PA, USA

Corresponding Author

Kimimasa Tobita, M.D.

Rangos Research Center, room 8121

4401 Pennsylvania Avenue,

Pittsburgh, PA 15224

Email Address: kit3@pitt.edu

Contents

Supplementary Figures

Supplementary Figure 1. Testing of Soluble Factors for Cardiomyocyte Induction

Supplementary Figure 2. Artificial Muscle Tissue (AMT)

Supplementary Figure 3. Cardiac Gene Expression of iPS Cell Derived Cardiomyocytes Compared to 4F-AG-AMT

Supplementary Movie 1. Video of spontaneously beating AMT.

Supplementary Movie 2. Intracellular Calcium Transient in spontaneously beating 4F-AG-AMT.

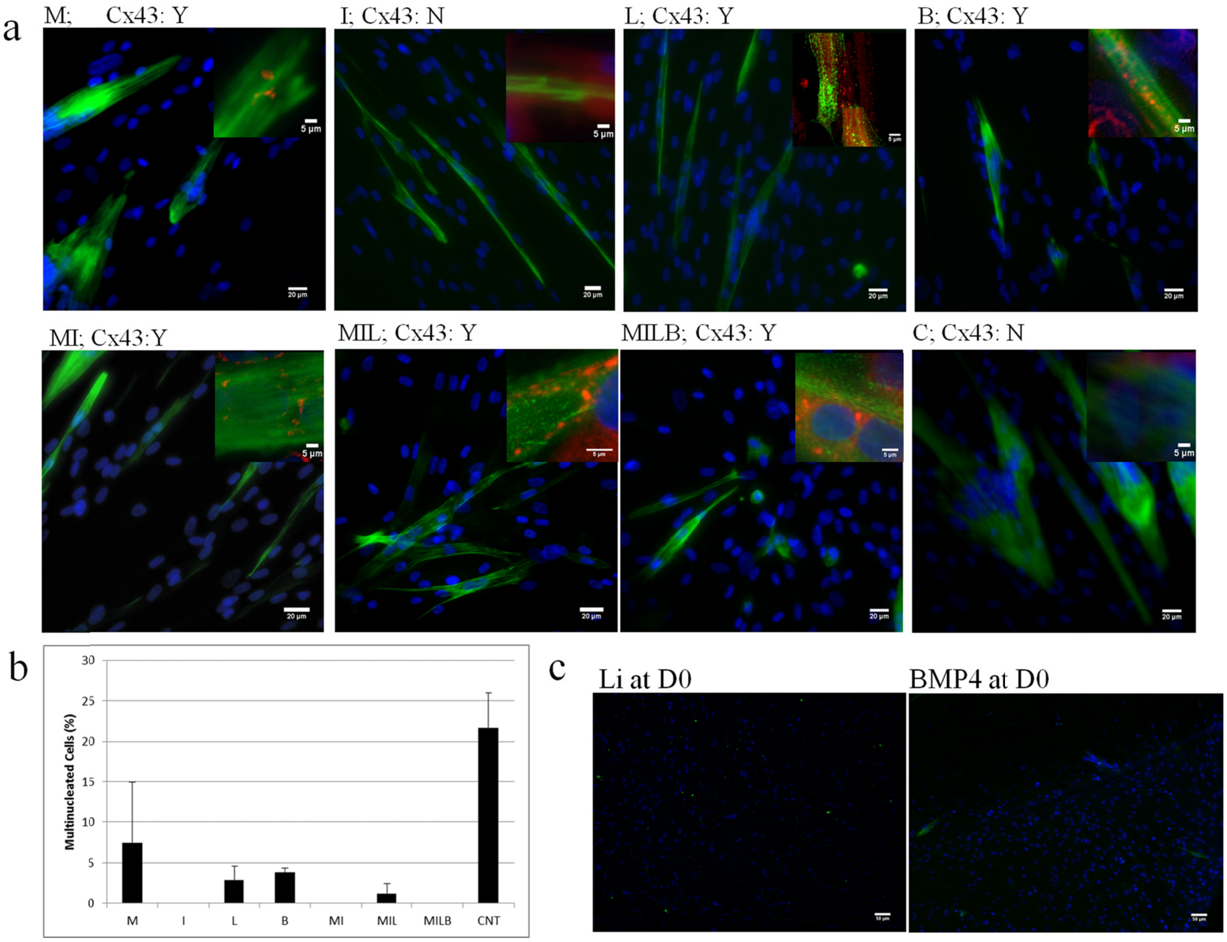

Supplementary Figure 1. Testing of Soluble Factors for Cardiomyocyte Induction. (a) Histological analysis of Chemical Induced MDSCs. MDSCs were seeded in chamber slides. Compounds were applied individually or in combination at the times specified in the 4F-AMT protocol. Cells were stained for TNNT2 (green) and TNNT2/Cx43 (insert frame) to assess muscle differentiation and gap junction formation. DAPI (blue) indicate nuclei. (b) Effects of soluble compounds on myotube formation. The percentage of nuclei in multinucleated TNNT2+ myotubes under each condition was quantified (n=3). (c) Effect of early LiCl and BMP4 treatment. Treatment of MDSCs with LiCl or BMP4 at day 0 of differentiation delayed differentiation assessed by α-Actinin expression at day 7. Abbreviations: M: miR-206 inhibitor; I: IWR-1; L: Lithium Chloride; B: BMP-4; CNT: Control; Y: Yes; N: No.

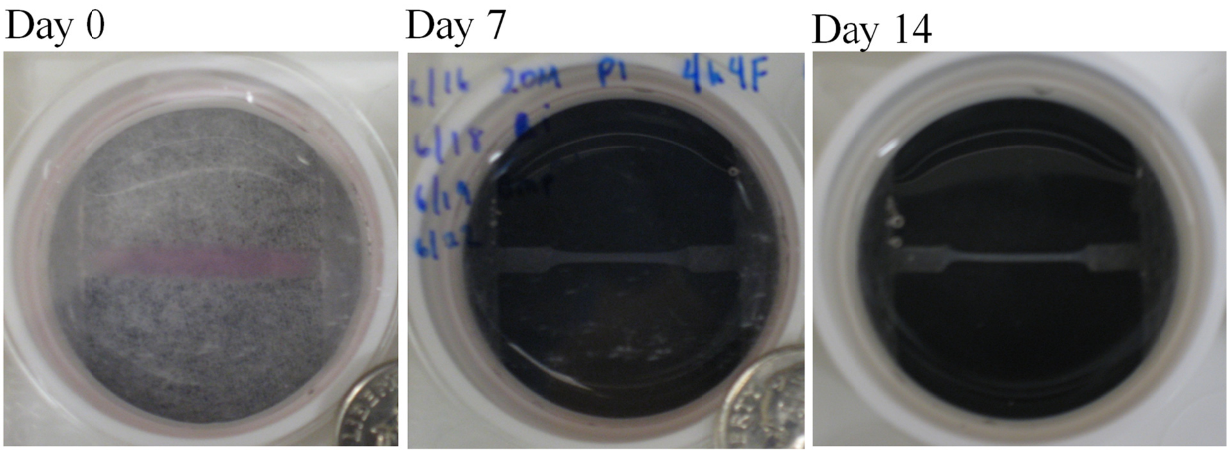

Supplementary Figure 2. Artificial Muscle Tissue (AMT) is formed as a linear construct and undergoes shrinkage over the course of the 14 day culture period. Well diameter is 34.8mm.

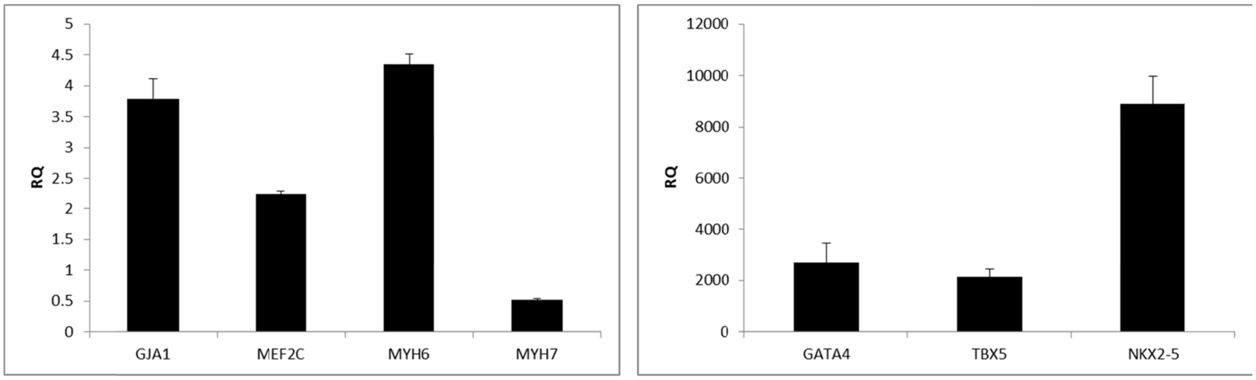

Supplementary Figure 3. Gene Expression of iPS Cell Derived Cardiomyocytes compared to 4F-AG-AMT. Values are expressed as fold change in gene expression and normalized to 4F-AG-AMT.
